# Barriers to Meeting the Social and Emotional Needs of Nursing Home Residents

**DOI:** 10.1093/geroni/igaa057.2534

**Published:** 2020-12-16

**Authors:** Amy Restorick Roberts, Mercedes Bern-Klug

**Affiliations:** 1 Miami University, Oxford, Ohio, United States; 2 University of Iowa, Iowa City, Iowa, United States

## Abstract

Presenters will describe barriers to psychosocial care and identify which factors increase the odds of experiencing a psychosocial care barrier. Reported major barriers include: insufficient number of nurse aide staff (31%), having to do things other people could do (29%), lack of resources to provide residents with opportunities to leave the nursing home on outings (25%), pressure to admit and discharge post/sub-acute patients takes time away from attending to the social and emotional needs of long stay residents (23%), and not enough social service staff for the number of residents (21%). With data from the 2019 National Nursing Home Social Services Directors Survey, a series of logistic regressions found that significant predictors varied by specific barrier, although devoting more time to short-term residents predicted a greater likelihood of reporting a major barrier in four of the five outcomes. Strategies to address these structural and contextual factors will be discussed.

